# Architects and Modern Hospitals

**Published:** 1900-04-14

**Authors:** 


### The Institutional Workshop.

### ARCHITECTS AND MODERN HOSPITALS.

In view of the very large amount of money which is being spent, and the still larger amount which promises to be spent within the next few years in the construction of hospitals of one sort and another, it seems a not altogether creditable, and certainly a very undesirable, state of affairs that there should be such differences among architects of repute in regard to many of the principles of hospital construction as appear to exist. According to the purposes for which a hospital is to be used—according, that is, as it is to be a general, a special, or an isolation hospital, certain radical differences will, of course, be found in the general plan, in the internal arrangements, and perhaps even in the materials used. But to whichever of these classes the hospital required may belong, the climate to be provided against is practically the same, the patients are the same, the treatment they require is the same, the funds from which the hospital is to be supported are the same, and the necessity for economy is almost the same in whatever part of England it may be placed; and it does not seem unreasonable to ask that some approach to a uniform standard and to uniform methods of attaining that standard should be accepted among architects as expressing what is regarded as appropriate in hospital construction. We are led to make these remarks by an incident which recently occurred.

A few weeks ago Mr. William Henman, F.R.I.B.A., well known as the architect of the Birmingham General Hospital, read a paper at the Carpenters' Hall on Modern Hospitals. We made a brief reference to this lecture in The Hospital, but lack of space prevented us from dealing with it in greater detail at the time. Some time afterwards we received a letter from a well-known architect criticising this lecture in the most stringent terms. We are not at liberty to mention this gentleman's name, but our readers may take it from us that it is, as the Americans would say, "high up" in the architectural world, and is especially well known in reference to hospital construction. The lecture, however, had by this time become stale news, and, as the letter had to be treated as anonymous, the whole thing appeared at first to be without journalistic interest. But on comparing the opinions of the lecturer and the critic on questions of the greatest importance in hospital construction, the feeling became strong that in points like these, affecting as they do the expenditure of large sums of public money every year, some greater unanimity ought to be arrived at than seems to exist among men whom we may properly call leading men in their profession. For example, the lecturer says:

Apart from the broad treatment in architectural designs of hospitals there are minor matters of detail worthy of attention. For instance, sanitary conveniences are required throughout hospitals, therefore soil and waste pipes are a necessity, and have to be provided in numerous and frequently prominent positions. In some cases they are hidden away in such a manner as to be difficult of access for inspection or repair, but more frequently, nowadays, it is the custom to exhibit on the exteriors of hospital buildings elaborate examples of the plumber's art. Yet I venture to believe you would give preference, so far as appearance is concerned, to the inoffensive arrangement of such pipes at the General Hospital, Birmingham, and judging from results there is not the least reason to suppose that the unsightly exhibition of the bare bones of plumbing requisites are one whit more serviceable, and certainly no less costly, nor more easily kept in suitable repair.

Commenting upon this the critic says:

It is somewhat difficult to understand from the lecture what Mr. Henman's special views as to the arrangement of soil and waste pipes may be; the only thing we can gather with any certainty is that the work at the General Hospital, Birmingham, is vastly superior to any other. This may or may not be, but if Mr. Henman means to imply that it is an unnecessary and undesirable thing to expose the system of pipes, not for waste water only, but also for service, then all we can say is that no one practically acquainted with the working of a hospital will agree with him. There is nothing to be ashamed of in an exposed pipe, as Mr. Henman seems to suppose; and the mere question of unsightliness is as nothing when weighed in the balance against the all importance of ready access.

On the question of walls and floors the lecturer says:

I must warn you against an error into which some have fallen by supposing that all walls, floors, and ceilings should be of hard and impervious materials. If so constructed rooms or wards are far from being pleasant places of abode, it is more difficult to properly temper and ventilate them, condensation takes place upon the more exposed surfaces in cold weather, sounds reverberate in a distressing manner, and being unyielding in their nature, such materials are liable to crack and rather difficult to repair or make good. Some suppose such buildings to be fire-resisting in the highest degree, but experiments demonstrate that materials more pervious have greater fire-resisting qualities. Although advocated by many authorities, it is to my mind very questionable whether it is advisable to coat all walls and ceilings with a non-pervious material in buildings, where natural means or exhaust methods of ventilation are depended upon. Doubtless the lower portions of walls, where they are liable to become dirtied or splashed, should be coated with a washable material, and be frequently wiped over. The floors also should be of more porous material. Teak or oak boarding, tongued and grooved, grounded with paraffin, wax, and wax polished, makes a most comfortable and at the same time a most efficient flooring. It can be kept clean without having to be wetted, and is less hard, cold, and unyielding than marble terrazzo, now frequently employed. That material, however, is perhaps the best available for corridors, operating, and such-like rooms; but when employed over large surfaces its unyielding nature causes irregular cracking, which cannot be satisfactorily repaired.

On this the critic comments as follows:

On the subject of wall and floor surfaces we are told, "I must warn you against an error into which some have fallen by supposing that all walls, floors, and ceilings should be of hard and impervious material." What is the argument on which this remarkable statement is founded? "If so constructed, rooms or wards are far from pleasant places of abode. It is more difficult properly to temper and ventilate them, condensation takes place upon the more exposed surfaces in cold weather, sounds reverberate in a distressing manner, and, being unyielding in their nature, such materials are liable to crack, and are then difficult to repair and make good." Truly a precious farrago of nonsense on which to found an indictment against a well-established principle founded on experience, and not like Mr. Henman's views, on pure imagination.

If there is one fact that is well established and recognised by long practical experience it is that permeable materials for the walls, floors, and ceilings of wards are certain sooner or later to become saturated with septic organisms, a condition fraught with danger to the patients. Long experience has taught that all these surfaces should be as cleanable and therefore as impermeable as possible. To say that wards constructed in this way are far from pleasant places of abode, that sounds reverberate in a distressing manner, is a ridiculous misstatement founded on absolute ignorance. That the exact converse is the fact anyone interested in the subject can satisfy himself by a visit to any recent hospital where the principles condemned by Mr. Henman are carried out.

Equally groundless is the assertion that walls or floors of impervious materials are liable to crack and cannot be repaired, and that ventilation is more difficult in wards so constructed. That the liability to crack if proper precautions are not used exists is undoubtedly true, but it is equally true that it is quite possible to guard against such a liability. So with ventilation. If it be as difficult, as Mr. Henman assumes, to ventilate a room lined with impervious surfaces, why in the name of commonsense did he line the walls of the Out-patient Waiting Hall at Birmingham with glazed faïence ware and the floor with marble? Of all departments of a hospital the out-patient waiting hall is the one which needs the most efficient ventilation, and this is the very place he chooses to construct in a way which he stigmatises as erroneous.

With regard to floors we are told that "teak or oak, grounded with paraffin and wax polished, makes the most comfortable, and at the same time the most efficient flooring." Here again is an assertion which can only be explained on the supposition of the lecturer's entire ignorance of the actual facts. The truth is that a wax-polished oak or teak floor is, for the nurses, the most uncomfortable, most tiring floor that exists. And in the case of floors it is the nurses who ought to have the first consideration, inasmuch as they are the people who make most use of them. There is no difference of opinion amongst those who have had practical experience of both kinds of flooring that terrazzo is a distinctly superior material than teak or oak. The theory that terrazzo is cold vanishes under practical tests, and among nurses who have had experience of both there is only one opinion as to their relative comfortableness.

He further adds, referring to Mr. Henman's remarks upon ventilation:

As might be expected, a lecture from such a source could not be completed without a reference to the so-called "Plenum" system of ventilation. We do not propose to discuss this question again. Our views on the subject have been expressed more than once, and nothing that we have seen at Birmingham or elsewhere has induced us to modify them. The interesting point however is that, judging from Mr. Henman's remarks about the plans for the New Royal Victoria Hospital, Belfast, he has at last realised the fact that a mechanical system of warming and ventilation, if thoroughly carried out, makes it possible to economise very considerably both in the size of the wards and the length of corridors. It is the one advantage, in fact, in a system which, as applied to wards, is, in our view, inappropriate and potentially dangerous.

We will not attempt at the present moment to criticise either the lecturer or his critic, but will content ourselves with saying that sanitary authorities and hospital managers who are compelled to spend money in hospital building, and, not unnaturally, desire to spend it to the best advantage, and in such a way as to secure accommodation of the most approved construction, are of necessity much embarrassed when they find that among architects of eminence such radical differences exist as to such elementary matters as the placing of drain-pipes, the construction of walls, the character of flooring, and the manner of ensuring ventilation.

